# Assessment of Minor Blood Group System Antigens and Their Phenotype among Voluntary Blood Donors in Ethiopian Blood and Tissue Bank Service, Addis Ababa, Ethiopia

**DOI:** 10.4314/ejhs.v33i5.11

**Published:** 2023-09

**Authors:** Getu Jenbere, Fekadu Urgessa, Melatwork Tibebu

**Affiliations:** 1 Ethiopian blood and tissue bank service, Addis Ababa, Ethiopia; 2 Department of Medical Laboratory Science, College of Health Science, Addis Ababa University, Addis Ababa, Ethiopia

**Keywords:** Minor blood group antigens, phenotype

## Abstract

**Background:**

Red blood cell antigens are numerous in structural and functional diversity; some are proteins while others are carbohydrates. The international society of blood transfusion currently recognized 43 blood group systems containing 349 red cell antigens. It also acknowledged 9 blood group systems (ABO, Rhesus, Kell, Duffy, Kidd, MNS, P, Lewis, and Lutheran) that are clinically significant and associated with hemolytic transfusion reactions as well as hemolytic disease of fetuses and newborns. The objective of this study was to assess the distribution of minor blood group antigens and their phenotype among voluntary blood donors in Ethiopian blood and tissue bank service in Addis Ababa.

**Method:**

A cross-sectional study was conducted from January to March 2022 among 260 volunteer blood donors to determine minor blood group antigens and their phenotype at EBTBS, Addis Ababa, Ethiopia. Tests were performed using Galileo Neo Immucor, which is fully automated Immunohematology analyzer.

**Result:**

A total of 260 blood donors were screened of which 153 (59%) were males. The antigen frequencies of minor blood group systems were: Fy(a), Fy(b), Jk(a), Jk(b), k, S, s were 33.5%, 43.5%, 97.7%), 40.4%), 100%, 45%, 90%, respectively. Regarding phenotype distribution, the most common phenotypes were: Duffy Fy (a-b+) 36.9%, MNS S-s+ 55% and Kidd Jk (a+b-) 59.6%.

**Conclusion:**

This study highlights the frequencies of Fy(a), Fy(b), Jk(a), Jk(b), k, S and s blood group antigens and their phenotypes in volunteer blood donors at EBTBS, Addis Ababa. For the management of alloimmunization cases in transfused patients, knowledge of these minor blood group antigens is relevant.

## Introduction

Red blood cells (RBC) antigens are inherited biological characteristics that do not change throughout life in healthy people (*1*). These RBC antigens are numerous in structural and functional diversity, some of them are proteins, glycoproteins, glycolipids, or carbohydrates *(2*). Depending on the type of antibody, which develops naturally in individuals or is formed as part of the immune response after exposure to foreign antigens; it can cause acute and delayed hemolytic transfusion reactions (HTR) (3). The international society of blood transfusion (ISBT) currently recognized 43 blood group systems containing 349 red cell antigens (4). In addition, around 9 blood groups which are clinically significant and associated with HTR and HDFN were also acknowledged by ISBT (5). These blood group systems are ABO, Rhesus, Kell, Duffy, Kidd, MNS, P, Lewis, and Lutheran (*6*). Next to ABO and Rh blood group systems, the Kell, Kidd, Duffy and MNS blood group systems are clinically important and may develop immune antibodies when exposed to corresponding antigens that can cause HTR secondary to fastest destruction of a significant number of transfused erythrocytes (7).

The major drawback of blood transfusion is the risk of developing antibodies against one or more erythrocyte antigens resulting from genetic differences between donor and recipient (8). Knowing minor blood group antigen would be significant for preventing alloimmunisation in young females, pregnant women, and patients who are expected to require repeated transfusions in life by providing them with antigen matched blood. Besides, antigen negative blood can be made available without delay to already alloimmunized multitransfused patients (9).

Immune antibody formation against red cell antigens is a common complication of transfusion reaction. Transfusing phenotype-matched blood can prevent the development of these antibodies. Clinically significant antibodies are capable of accelerating destruction of the corresponding antigen, so screening of donated blood for clinically significant minor blood group antigen before transfusion is essential to reduce transfusion reaction and ensure safe blood transfusion. However, in developing countries, especially in Ethiopia, transfusing ABO and Rh-compatible blood without screening clinically significant minor blood group antigens and their phenotype is a common practice.

This study may provide input significant for health personnel, officials working on transfusion activities and blood safety, non-governmental organizations and others who are engaged in blood transfusion activities. The findings can also be significant for those involved in improving the safety of blood and establish guidelines on minor blood group antigen and their phenotype. It may also be used as a baseline for researchers, and offers valid contribution to EBTBS and Federal Ministry of Health (FMoH).

## Methods and Materials

The study was conducted from January 2022 to March 2022 at EBTBS in Addis Ababa, Ethiopia. The Ethiopian Blood Bank (EBB) was established in 1962 by the Ethiopian Red Cross Society (ERCS) with support or assistance of an Israeli physician Dr. Ceril Levine. Since January 25, 2022, the blood bank has been re-established as an EBTBS under Regulation number of No. 1263/2021. EBTBS tasks are to coordinate, giving logistic and technical support as well as overseeing all the 43 regional blood banks, as well as managing and controlling tissue and organ transplantation throughout the country (10).

A cross-sectional study design was used to determine the prevalence of minor blood group antigens and their phenotypes among volunteer blood donors. To select blood donors, a convenient technique was applied. Thus, donors available during our sampling collection period were considered as source population whereas those who were eligible for the study and passed the inclusion criteria were our study population.

A total of 260 voluntary blood donors were participated in this study from January 2022 to March 2022 using convenient sampling technique. Age group of 18 to 65, weighing more than 45 kilograms and having normal hemoglobin values (For Male >13g/dl and females >12g/dl) were included in this study.

Questionnaire was used to collect profiles such as age, sex, and address, any history of the previous transfusion and drug intake. The general health conditions of blood donors, jaundice, and any clinically significant diseases related to autoimmune disorders were assessed through medical examination.

The study was granted ethical clearance by Addis Ababa University, Department of Medical Laboratory Science and Official Permission from EBTBS. During data collection, blood samples were labeled only with the donor identification number used as a unique study code. Confidentiality of the result was maintained by the authors of the study, and the results were only shared with the medical staff of authorized members of the study. Computerized files were password protected, and paper files were also locked safely being only accessible to authorized personnel.

**Laboratory procedure**: Blood samples were collected using an acceptable phlebotomy technique, by well-trained nurses, from a mobile campaign, and at the center clinic. A 5ml of blood sample was collected at the time of donation into a tube containing ethylenediaminetetraacetic acid (EDTA). From the mobile collection site, blood specimens were transported to the blood bank by maintaining the cold chain at 2–10°C with a cold box. After arrival at the blood bank, the blood samples were centrifuged at 3000 rpm for 5 min.

Blood samples were tested for minor blood group antigens and their phenotype using Galileo Neo Immucor Gamma automated system, fully automated Immunohematology analyzer that uses Capture-R Select (SPRCA-Solid Phase Red Cell Adherence). The Fy(a), Fy(b), Jk(a), Jk(b), S, s, and k antigens were determined by commercially prepared polyclonal (Immucor Med, Diagnostik GmbH, Germany) antisera. Capture -R select microwells contain only RBC binding agents, which allows to create RBC monolayers from any sample. The Capture -R Select Assay process includes two parts: monolayer preparation and assay processing. The RBC sample to be tested is added to the Capture -R Select well along with a drop of saline. The wells were centrifuged to enhance the binding of the cells at the bottom of the well. The supernatant and extra cells were washed out of the well leaving behind a monolayer of the cells to be tested. The reagents were added to wells, incubated at 370c, washed, Capture- R Ready indicator red cells added, centrifuged, and the result was read and interpreted (the Immunohematology analyzer Manual).

**Quality assurance and data analysis**: Blood samples were collected by well-trained and experienced nurses in EBTBS after getting written or oral informed consent from the blood donors. Universal precautions were taken during sample handling, processing, and testing. The kit manufacturer (Immucor, Inc, Germany) recommendations and EBTBS standards were followed for reagent storage and labeling. All data recordings were checked for completeness, and the results were recorded with the donor identification number.

Daily quality control of reagent antisera was performed using selected antigen positive and negative red cells (supplied by company) to confirm the reactivity and specificity. These reagents were tested with the corresponding antigen-positive and antigen-negative red blood cells. The reagents were considered appropriate for use if only antigen-positive red blood cells demonstrate a positive result.

The collected data was cleaned, coded, summarized, entered into an Excel spreadsheet, and imported to the Statistical Package for Social Science version 26 (SPSS Inc. Chicago, IL, USA) software for analysis and interpretation. The Data were analyzed using descriptive statistical analysis utilizing absolute frequencies and percentages, and the results were organized in tables and figures.

**Operational definition**: For this study, the antigen frequency of minor blood group Duff, Kid, Kell and MNS; and for the phenotyping the frequency of these minor blood group phenotypes was assessed.

## Results

A total of 260 donor blood samples were tested for minor blood group antigens and their phenotype. In this study, 58.8% (153/260) of the participants were males while 41.2% (107/260) were females, showing male predominance over females. The most common blood donors were the younger age group (18-25 years) who contributed about 61.9% (161/260) of the total blood donations. The mean age of the donors was 26 years, the median age was 22 years and the most frequently donating age group was 18 years.

The frequencies of clinically significant minor blood antigens Fy(a), Fy(b), S, s, Jk(a) and Jk(b) among donors were 33.5%, 43.5%, 45%, 90%, 97.7% and 40.4%, respectively. Concerning the Kell blood group system, the k antigen was found in 100% of the donors. No donor was typed as Cellano (k) negative; therefore, this antigen can be labeled as “dominant” in Ethiopian (Table 1).

**Table 1:** Frequency of clinically significant minor blood grouping antigens with sex at EBTBS

Blood Group System	Antigen	Male		Female		Total	

		frequency	percent	frequency	percent	frequency	percent
Duffy(a)	Fy(a+)	49	32.0%	38	35.5%	87	33.5%
	Fy(a-)	104	68.0%	69	64.5%	173	66.5%
Duffy(b)	Fy(b+)	69	45.1%	44	41.1%	113	43.5%
	Fy(b-)	84	54.9%	63	58.9%	147	56.5%
S	S+	73	47.7%	44	41.1%	117	45.0%
	s-	80	52.3%	63	58.9%	143	55.0%
s	s+	137	89.5%	97	90.7%	234	90.0%
	s-	16	10.5%	10	9.3%	26	10.0%
Kidd(a)	Jk(a+)	150	98.0%	104	97.2%	254	97.7%
	Jk(a-)	3	2.0%	3	2.8%	6	2.3%
Kidd(b)	Jk(b+)	64	41.8%	41	38.3%	105	40.4%
	Jk(b-)	89	58.2%	66	61.7%	155	59.6%
Kell(k-li)	k+(Cellano +)	153	100%	107	100%	260	100%

Concerning phenotype distribution, the most common Duffy phenotype encountered in this study was Fy(a-b+) 96(36.9%) followed by phenotype Fy(a-b-) 77(29.6%), Fy(a+b-) 70(26.9%) and the least common phenotype was Fy(a+b+) 17(6.5%). As for the MNS blood group system, S-s+ was the most common phenotype at a frequency of 143(55%) followed by phenotype S+s+ 91(35%), S+s- 26(10%). In the Kidd phenotype system, the Jk(a+b-) phenotype was the most prevalent, observed in 155(59.6%) of the total samples followed by the Jk(a+b+) 99(38.1%), Jk(a-b+) 6(2.3%) individuals of the total population. The null phenotypes S-s- and Jk(a- b-) were not detected in any donor sample. The antigen frequencies of the Duffy blood group system were Fy(a) 33.5% and Fy(b) 43.5%. The frequency of major Duffy antigen phenotypes in this study was; Fy(a-b+): 36.9%, being the most common phenotype in our blood donor population (Table 2).

**Table 2:** Frequency of Duffy, MNS, Kidd, and Kell Blood Group Phenotypes at EBTBS (n=260)

Blood Group System	Phenotype	Male		Female		Total	

		frequency	Percent	frequency	Percent	frequency	Percent
Duffy(Fy)	Fy(a+b+)	10	3.8%	7	2.7%	17	6.5%
	Fy(a+b-)	39	15.0%	31	11.9%	70	26.9%
	Fy(a-b+)	59	22.7%	37	14.2%	96	36.9%
	Fy(a-b-)	45	17.3%	32	12.3%	77	29.6%
MNS	S+s+	57	21.9%	34	13.1%	91	35.0%
	S+s-	16	6.2%	10	3.8%	26	10.0%
	S-s+	80	30.8%	63	24.2%	143	55.0%
Kidd(Jk)	Jk(a+b+)	61	23.5%	38	14.6%	99	38.1%
	Jk(a+b-)	89	34.2%	66	25.4%	155	59.6%
	Jk(a-b+)	3	1.2%	3	1.2%	6	2.3%
Kell(K-li)	k+(Cellano +)	153	58.8%	107	41.2%	260	100.0%

## Discussion

The study identified the clinically significant minor blood group antigens and their phenotype distribution among voluntary blood donors. The frequencies of clinically significant antigen Fy(a), Fy(b), S, s, Jk(a) and Jk(b) among donors were 33.5%, 43.5%, 45%, 90%, 97.7% and 40.4%, respectively, whereas the k antigen was found in 100% of donors (Table 1). No donor was typed as Cellano (k) negative. Therefore, this antigen can be labeled as “dominant” in Ethiopian, which was similar to that of studies done in the Indian, Saudi Arabia, African, and Chinese blood donor populations which had 100% prevalence rate (11, 12, 13, 14). It is also comparable with study findings by Al-Riyami AZ, *et.al*, among Omani blood donors (15) and by Randa M in Egyptian population (16), showing 99.4% and 99% prevalence, respectively. The antigen frequencies of the Duffy blood group system in Ethiopian blood donors at EBTBS were; Fy(a) 33.5% and Fy(b) 43.5%, which had significant differences from other ethnicities, i.e., Indians, Caucasian, African, Chinese, Saudi Arabia, Egyptians and United Arab Emirates populations (11, 12, 13, 14, 15, 17), as shown in Table 3. This may be because of study population, sample size and immunohematology analyzer variation.

**Table 3:** Comparison of Prevalence of minor blood group antigens in blood donors of EBTBS with other **ethnic groups**

Antigen	Current Study		Indian (20)	Caucasian (23)	African (23)	Chinese (24)	Egyptians (25)	Saudi Arabia (21)
Fy(a)	87	33.5%	87.4%	66.0%	10%	99%	26.7%	22%
Fy(b)	113	43.5%	57.7%	83.0%	23%	9.2%	48.9%	22%
S	117	45%	54.8%	55.0%	31%	8.7%	56.8%	59%
s	234	90%	88.7%	89.0%	93%	100%	86.1%	83%
Jk(a)	254	97.7%	81.4%	77.0%	92%	73%	83.9%	86%
Jk(b)	105	40.4%	67.6%	74.0%	49%	76%	58.6%	60%
k	260	100%	100%	98.8%	100%	100%	99%	100%

The prevalence of major Duffy antigen phenotypes in this study was; Fy(a-b+): 36.9%, being the most common phenotype in our blood donor population, comparable with White people's (34%). The frequencies of this phenotype show variation between ethnic groups, as shown in Table 4. The prevalence of Fy(a-b+) phenotype of our study was higher than Indian, African, Chinese, and Omanis blood donors, with the frequencies of 12.3%, 22%, 0.3%, and 14.9%, respectively. The other homozygous phenotype Fy(a+b-) was the 3^rd^ predominant phenotype, observed in 26.9% of donors, which was greater than the observed frequencies in White (17%), Africans (9%), and Omanis (9.2%) populations. Conversely, this frequency was relatively low compared with Indian (42.1%), and Chinese populations (91%). The heterozygous phenotype Fy(a+b+) was detected in 6.5% of our blood donors, making it the least prevalent phenotype. The frequency of Fy(a+b+) in the Indian, Omanis, African, and Chinese blood donor population also shows comparable results, which were; 4.5%, 7.4%, 1%, and 8.9%, respectively. However, it was relatively high in the White population 49% (11, 13, 14, 16). The null phenotype Fy(a-b-) was the 2^nd^ most common phenotype, which was observed in 29.6% of donors. Remarkably, it was significantly lower than in the African (68%) and Omanis (68.5%) population as well as higher than in India (0.3%). This null phenotype is absent in Chinese and rare in White populations (11, 13, 15).

**Table 4:** Frequencies of phenotypes for Duffy, MNS, and Kidd systems of blood donors of EBTBS with other ethnic groups

Phenotype	Current Study	Indian (20)	Whites (20)	African (23)	Chinese (24)	Omanis (22)
Duffy						
Fy(a+b+)	6.5%	4.5%	49%	1%	8.9%	7.4%
Fy(a+b-)	26.9%	42.1%	17%	9%	91%	9.2%
Fy(a-b+)	36.9%	12.3%	34%	22%	0.3%	14.9%
Fy(a-b-)	29.6%	0.3%	very rare	68%	0	68.5%
Ss						
S+s+	35%	43.9%	44%	28%	8.7%	46.1%
S+s-	10%	11.3%	11%	3%	0	17.9%
S-s+	55%	44.7%	45%	69%	100%	36%
S-s-	0	very rare	very rare	1%	very rare	0
Kidd						
Jk(a+b+)	38.1%	48.9%	49%	34%	49.1%	47%
Jk(a+b-)	59.6%	32.5%	28%	57%	23.2%	35.4%
Jk(a-b+)	2.3%	18.5%	23%	9%	26.8%	17.3%
Jk(a-b-)	0	2%	very rare	very rare	0.9%	0.3%

The antigen frequencies of MNSs blood group system in our study S and s antigens were 45% and 90%, respectively, and almost comparable with the study done in Egypt (S=56.8% and s=86.1%) (16), India (S=54.8% and s=88.7%) and Caucasian population (S=55% and s=89%). A higher prevalence of the S antigen was observed compared to what has been reported in Africa and Chinese, but the s antigen was comparable with all other studies (Table 3). In this study, the prevalences of major MNSs antigens phenotypes were; S-s+; 55%, being the most common phenotype in our blood donors. The frequencies of this phenotype vary between ethnic groups, as shown in Table 4. In India, Whites, Africans, and Chinese and Omanis, the frequencies were 44.7%, 45%, 69%, 100%, and 36%, respectively. According to our study, S-s+ phenotype findings was higher than Indians, Whites, and Omanis ethnic groups, but lower than African (69%) and Chinese (100%) donor population. The heterozygous phenotype S+s+ was the second predominant phenotype, observed in 35% of donors, which was greater than the observed frequencies in African (28%) and Chinese (8.7%) populations (11, 12, 13, 14). Conversely, this frequency was relatively low compared to White (44%), Indians (43.9%), and Omanis (46.1%) donors. The homozygous phenotype S+s- was detected in 10%, making it the least prevalent phenotype comparable with studies done in Indian and Omanis populations, which were 11.3%, and 17.9% respectively (11, 15). However, this phenotype's frequency was high in the Chinese (0% and African populations (3%) (13, 14).

The prevalence of Kidd blood group antigens observed in this study were; Jk(a) 97.7% and Jk(b) 40.4%, much higher than the study done by Solanki A. *et al.* on blood donors of north India (Jk(a) 4% and Jk(b) 4.4%) (*18*); and also varies from study conducted in Saudis, Jazan Province by Owaidah et al (Jk(a) 86% and Jk(b) 60%) (12). This variation may be because of size of study population, ethnicity and geographical variation. The prevalence of Jk(a) in this study was higher than the one in studies done in India, Caucasians, Africans, Chinese, Egyptians, and Saudi Arabia with the frequency of 96.5%, 81.4%, 77%, 92%, 73%, 83.9%, and 86%, respectively. However, the prevalence of Jk(b) was comparable with African (49%), even though smaller than other findings: Indian (67.6%), Saudi Arabia (60%), Caucasian (74%), Chinese (76%), and Egyptians (58.8%) (11, 12, 13, 14, 16). This could be due to the geographic locations and the ethnicities of the blood donors.

Of the Kidd phenotypes, the most common Kidd blood groups system phenotype in this study was Jk(a+b-) 59.6%, relatively similar with that reported among the African populations at 57%. However, higher than studies done on Indians, Whites, Chinese, and Omanis who have the frequency of 32.5%, 28%, 23.2%, and 35.4%, respectively. On the other hand, the heterozygous phenotype Jk(a+b+) was the second predominant phenotype observed in 38.1% of donors, which was comparable to findings reported among the African populations at 34% and lower than that in the Indian (48.9%), Whites (49%), Chinese (49.1%) and Omanis (47%). The homozygous Jk(a-b+) phenotype was found in 2.3%, making it the least prevalent phenotype comparable with studies done in Indian, White, Omanis, and African populations of 9%, 18.5%, 23%, and 17.3%, respectively (11, 13, 15). Lastly, the null Jk(a-b-) phenotype was not detected in Ethiopian blood donors. These finding was consistent with studies conducted in the United Arab Emirates, North and South Indian donor population (17, 19, 20)

It was challenging to compare the results of different studies because of the heterogeneity of the populations' involved, varied screening protocols, and differences in the techniques used for antigen and antibody identification. Besides, the distribution of the blood group antigens among different racial-ethnic groups shows different findings.

The limitation of this study was that in the Kell blood group system, the K antigen and phenotyping (Kk) were not done because of lack of reagents at EBTB during the study period.

In conclusion, this study laid a foundation for minor blood group antigens and their phenotype frequency among voluntary donor in EBTBS, which could play an important role in developing knowledge to ensure safe blood transfusion. Besides, it may also help to build donor database of regular voluntary blood donors with known antigenic profile for specific population in needy, especially for antigen matched blood to young females, pregnant women, patients who are expected to require repeated transfusions in life and have developed unexpected antibodies.

Although this study was conducted on a small group of blood donors compared to our country's total population, still, it highlights the frequencies of minor blood group antigens. We recommend all stakeholders for a larger nation-based study involving blood donors from different regions in Ethiopia to confirm this finding. And also, to assess for any region-specific variations in clinically significant minor blood group antigens and their phenotype among blood donors or other population in the country anticipating the heterogeneity with geographical, ethnicity and genetic variation.
